# The Cationic Ring-Opening Polymerization of Tetrahydrofuran with 12-Tungstophosphoric Acid

**DOI:** 10.3390/molecules15031398

**Published:** 2010-03-08

**Authors:** Ahmed Aouissi, Salem S. Al-Deyab, Hassan Al-Shahri

**Affiliations:** Chemistry Department, College of Science, King Saud University, Riyadh, Saudi Arabia

**Keywords:** cationic polymerization, cyclic ethers, heteropolyacid, tetrahydrofuran, Ring-opening

## Abstract

The cationic ring-opening polymerization reaction of tetrahydrofuran at 20 °C was catalyzed by H_3_PW_12_O_40_·13H_2_O as solid acid catalyst. The effect of the proportions of acetic anhydride and catalyst, reaction time and support on the polymerization reaction was investigated. It has been found that the yield and the viscosity of the polymer depend on the proportion of acetic anhydride, the presence of the latter in the reactant mixture being required for the ring-opening. The catalytic activity of the alumina-supported heteropolyacid results showed that Brønsted acid sites are more effective than Lewis ones for the cationic ring-opening polymerization.

## 1. Introduction

Due to their high polarizability and flexibility, polyethers constitute a very important soft segment for producing thermoplastic elastomers such as polyesters (Hytrel^®^) and polyurethanes (Spandex). They represent a key ingredient in the production of a variety of elastomeric products. Therefore, they have been the subject of a large number of papers [1,2,3,4,5]. The polymerization for their production is initiated by electrophilic agents such as Brønsted acids (HCl, H_2_SO_4_, HClO_4_, *etc.*) and Lewis acids (AlCl_3,_ BF_3_.OEt_2_, TiCl_4_, *etc.*). However, the protonic acid catalysts used are very noxious and corrosive. As for Lewis acids, it is known that their use requires large amounts to achieve acceptable yields of polymers. Increasing environmental concerns in recent years have resulted in a demand for more effective catalytic processes. In this regard, studies have been carried out on the development of solid acids to replace aggressive and dangerous homogeneous acids to overcome the problems of separating the catalyst from the products and the disposal of solid/liquid wastes. 

Solid Brønsted acids with superacidic character, such as the Keggin-type heteropolyacids, are known as highly active catalysts [6,7,8,9,10,11]. Due to their strong acidity, these “superacids” catalyze various reactions much more effectively than the conventional protonic acids [12,13]. They have been found efficient for a variety of organic reactions [14,15,16]. In recent years, heteropolyacids have been used as catalysts to induce the polymerization of various monomers such as cyclic ethers, styrene, acetals, polyalcohols and lactones [17,18,19,20]. In our previous paper [21] we have reported the polymerization of tetrahydrofuran catalyzed by a series of heteropolyanions and initiated by acetic anhydride (AA). It was shown that 12-tungstophosphoric acid (12-HPW, H_3_PW_12_O_40_.13H_2_O) was the best catalyst among the series of heteropolyanion catalysts tested. This fact prompted us to investigate the cationic ring-opening polymerization of tetrahydrofuran (THF) by this efficient catalyst. The effect of AA proportion, reaction time, catalyst amount and support on the polymerization were investigated.

## 2. Results and Discussion 

### 2.1. Catalyst characterization

The identity of the synthesized H_3_PW_12_O_40_.13H_2_O was proven by comparison of its FTIR and thermogravimetric analysis data with those reported in literature [22]. The main characteristic bands of the Keggin structure are observed at 1,080–1,060 cm^-1^ (ν_as_ P-O_a_), at 990–960 cm^-1^ (ν_as_ Mo-O_d_), at 900–870 cm^-1^(ν_a_ Mo-O_d_-Mo), and at 810–760 cm^-1^ (ν_as_ Mo-O_c_-Mo). The number of hydrogen atoms and water molecules in the H_3_PW_12_O_40_·13H_2_O was determined by thermogravimetric analysis through the weight loss observed as the temperature is increased. Loss of crystallization water (13–14 H_2_O) was observed between 160 and 280 °C. Loss of the ‘constitutional’ water molecules, *i.e.* the protons bound to the polyanion external oxygens, are (1.5 H_2_O) was observed above 350 °C. This result is in agreement with published results [22].

### 2.2. Polymer characterization

**Figure 1:**
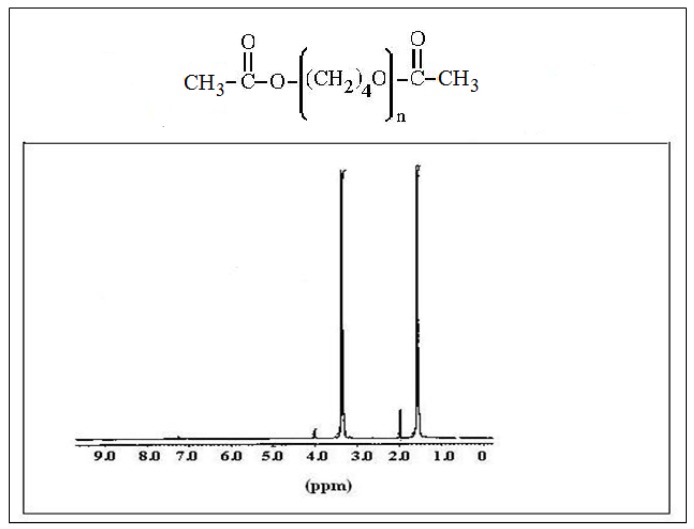
^1^H-NMR spectrum of poly(THF) in CD_3_OCD_3._

The polymerization of THF can be induced by Keggin-type heteropolyacids under mild conditions. As evidenced by ^1^H-NMR (Figure 1 and Table 1), the results showed that H_3_PW_12_O_40_·13H_2_O induced the polymerization of tetrahydrofuran. The number average molecular weight ($\bar{M_{n}}$) for the polymers can be calculated by integrating the repeat unit protons with the end-group protons on the basis of the integral data [23]: $\bar{M_{n}}$ = 102 + 72 *n.* It has been found that the values vary from 1,360 to 4,535 g/mol.

The ring opening polymerization proceeds via a cationic mechanism (Scheme 1). In this mechanism we assume that the protons carried by the heteropolyacid induce the polymerization. 

The first stage is the protonation of the acetic anhydride. The next stage is a nucleophilic attack of the oxygen of the THF on the carbocation of the chains in growth [24,25]. The presence of the acetate groups at the two ends of the chain was clearly identified by ^1^H-NMR, therefore the last stage must be a nucleophilic attack on the carbon located alpha to the positive charge bearing oxygen of the chains in growth by the oxygen of the acetic acid formed in the first stage from the protonation of acetic anhydride.

### 2.3. Polymerization 

In order to investigate the activity of Brønsted solid acid catalyst, H_3_PW_12_O_40_·13H_2_O for the polymerization of cyclic ether, we have investigated the effect of the AA proportion, the amount of the catalyst, the polymerization reaction time and the support on the polymerization reaction 

#### 2.3.1. Effect of AA proportion

**Figure 2:**
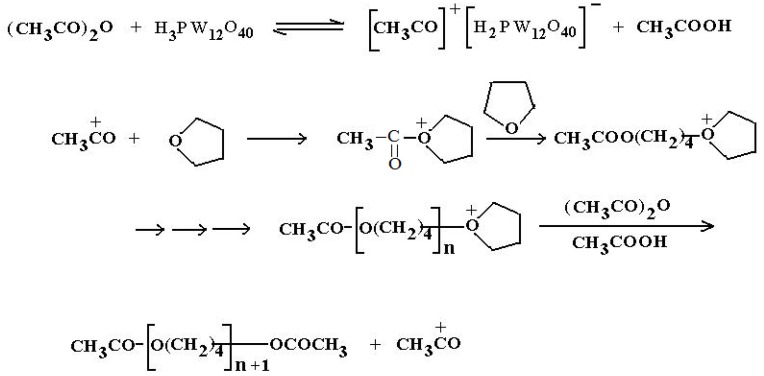
Mechanism of THF ring opening followed by polymerization.

The results of THF polymerization induced by 0.1 g of 12-tungstophosphoric acid in bulk during 1.5 h are reported in Figure 2 and Table 2. It can be seen from these results that the value of the conversion depends on the AA proportion. In fact, when the AA/THF volume ratio varies from 0 to 0.25 the conversion increases from 0% to 60%, then after that it decreases. The decrease of the conversion at high AA proportions might be due to consumption of the heteropolyacid protons by the acetic anhydride leading to acetic acid (a weak acid). In fact, when the reaction was performed without the catalyst but with acetic acid, no polymer was obtained. It is also noteworthy that the polymerization does not occur in the absence of AA. The decrease of the conversion at high acetic anhydride proportions is due to an increase in the number of methyl groups at the extremities of the chains which blocks the polymer chain growth.

If one takes into account simultaneously the conversion and the viscosity, one can see from the Figure 2 and Table 2 that the volume ratio of AA/THF equal to 0.2 is the optimum for the synthesis of poly(THF). In fact, when this later was performed with this ratio, the conversion is higher and the viscosity is the highest. This result has prompted us to select this ratio to study the effect of the reaction time, the catalyst amount and the catalyst support on the polymerization.

#### 2.3.2. Effect of reaction time

**Figure 3:**
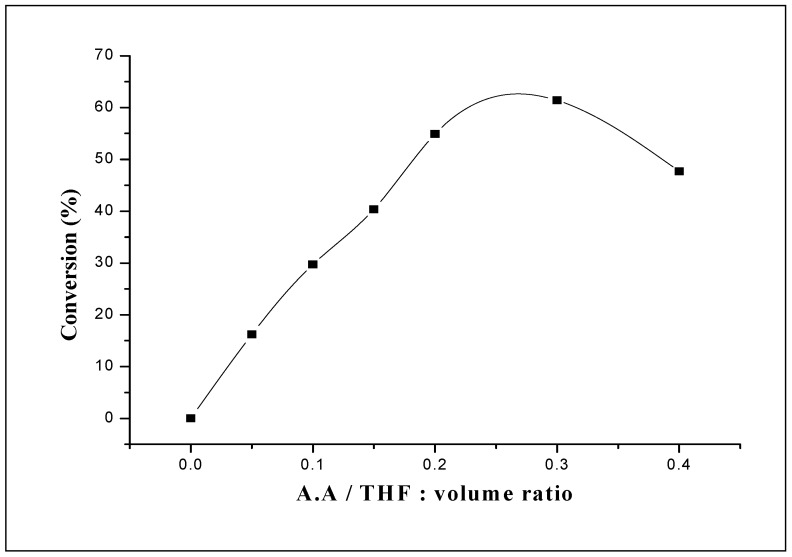
Effect of AA proportion on the yield of poly(THF) catalyzed by 0.1 g of H_3_PW_12_O_40_·13H_2_O. Reaction conditions: T = 20 °C; Reaction time = 1.5 h.

The result of the reaction time effect is depicted in Figure 3. It can be seen from the figure that the conversion increases with time up to two hours, then after the conversion remained almost stable with a conversion about ≈63%. 

#### 2.3.3. Effect of amount of catalyst

**Figure 4:**
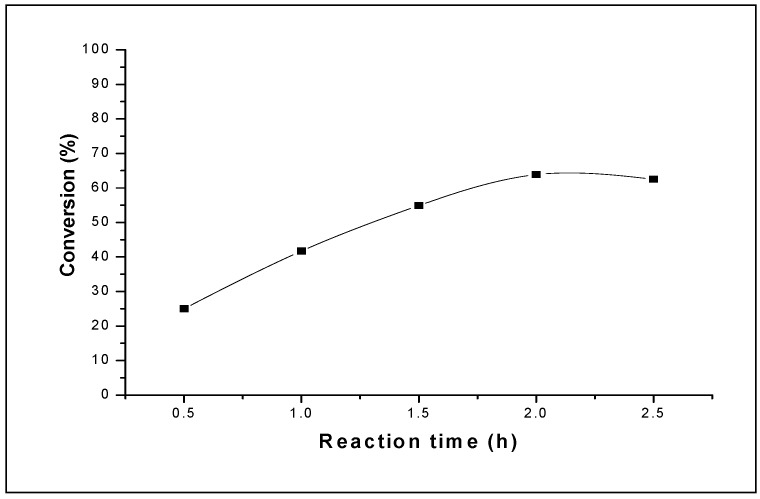
Influence of the reaction time on the conversion of THF catalyzed by 0.1 g of H_3_PW_12_O_40_·13H_2_O. Reaction conditions: T = 20 °C; AA/THF (volume ratio) = 0.2.

**Figure 5:**
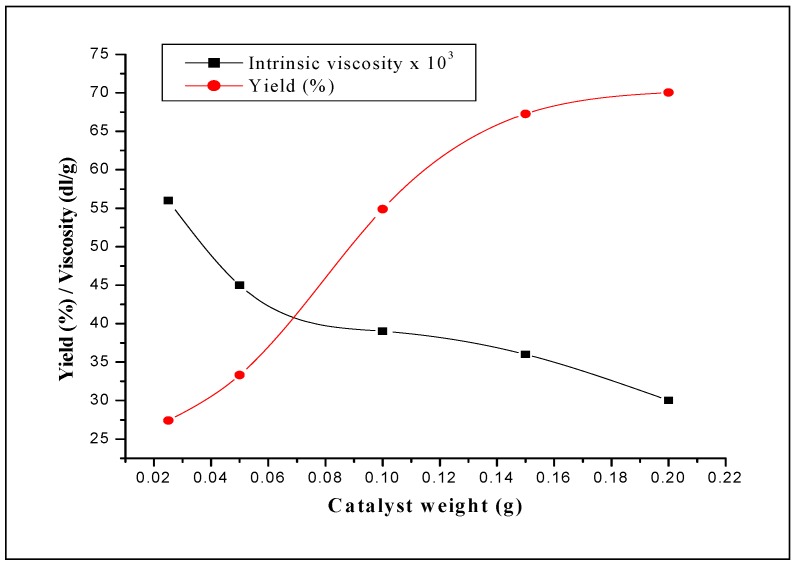
Effect of the amount of the catalyst on the conversion and on the intrinsic viscosity of the Poly(THF). Reaction conditions: T = 20 °C; AA/THF (volume ratio) = 0.2.

Figure 4 illustrates the results of the effect of the amount of the catalyst on the polymerization. It can be seen from this figure that the conversion increases as the amount of H_3_PW_12_O_40_·13H_2_O increases. This is probably the result of an increase in the number of initiating active sites responsible for inducing polymerization, which is proportional to the amount of catalyst used in the reaction. It can be seen also from this figure that when the amount of the catalyst increases, the conversion increases, whereas the intrinsic viscosity of the Poly(THF) decreases. In fact, when the amount of catalyst was varied from 0.025 g to 0.2 g, the conversion increased from 27.4% to 70.1% whereas the intrinsic viscosity decreased from 0.056 to 0.030 dL/g. It can be seen from Figure 4, that at higher amounts of catalyst, the conversion decreases slightly. The decrease of the intrinsic viscosity with increasing the catalyst amount can be explained by the fact that the monomer to initiator ratio is altered, i.e, fewer monomer units are available per propagating polymer. The number of initiating active centers responsible of inducing polymerization is prorate to the catalyst amount used in the reaction. Similar results were obtained by Yahiaoui *et al.* [26] in the polymerization of cyclohexene oxide by a montmorillonite sheet silicate clay, exchanged with proton, H-Maghnite (Brønsted solid acid catalysts). Like the molecular weight, the T_m_ decreases with the increase of the amount of catalyst (Figure 5). 

#### 2.3.4. Effect of the support (Al_2_O_3_)

**Figure 6:**
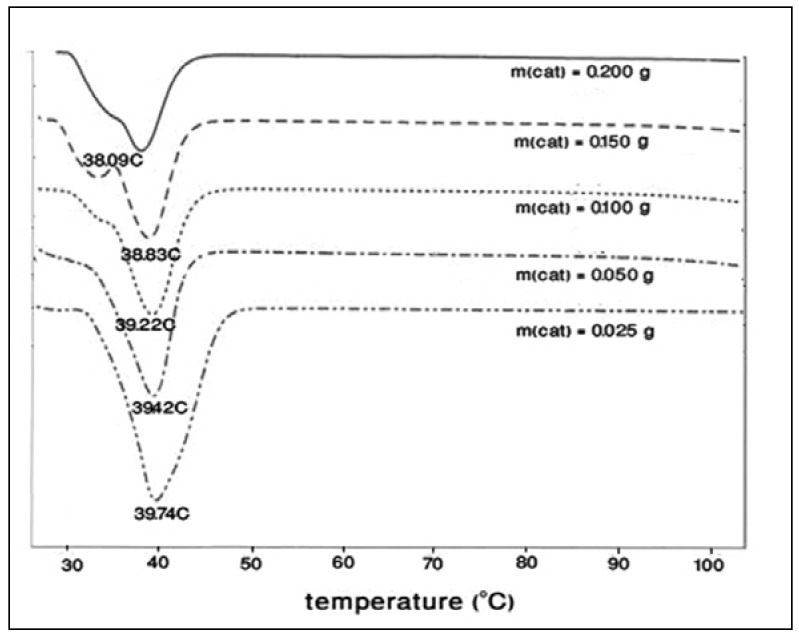
Effect the amount of the catalyst on the T_m_ of poly(THF). Reaction conditions: T = 20 °C; AA/THF (volume ratio) = 0.2.

The effect of the support was investigated using alumina as a support (Figure 6). The polymerization reactions were performed using various amounts of catalysts. By comparison of conversion values obtained on the unsupported and the alumina-supported heteropolyacid, it can be seen that the unsupported heteropolyacid is more active than the supported ones for all the amounts of catalysts tested. This results might be explained by the fact that in the alumina supported catalyst a certain number of protons are trapped through an heteropolyacid-support interaction and the number of Brønsted-acid sites decreases, inducing the decreases of the Brønsted-acid character leading to a decrease of the conversion [11,27,28].

**Figure 7:**
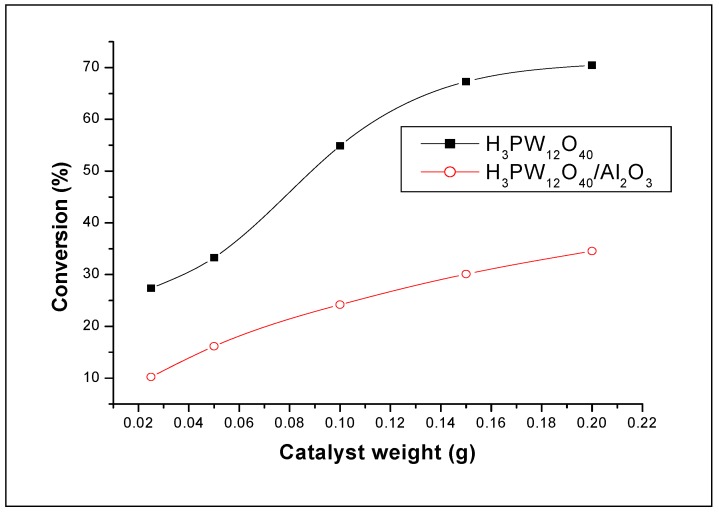
Values of the conversion obtained with the unsupported and the alumina-supported catalysts. Reaction conditions: T = 20 °C; AA/THF (volume ratio) = 0.2.

## 3. Experimental 

### 3.1. Preparation of the catalyst

H_3_PW_12_O_40_·13H_2_O was prepared according to the method described in the literature [29]. The alumina supported heteropolyacid was prepared by incipient-wetness of impregnation of γ-Al_2_O_3_ with aqueous solution of H_3_PW_12_O_40_·13H_2_O with concentration high enough to avoid its degradation [30]. 

### 3.2. Catalyst characterization 

The Keggin structure of 12-tungstophosphoric acid H_3_PW_12_O_40_·13H_2_O was characterized by infrared (IR) spectroscopy. IR spectra were recorded with an infrared spectrometer GENESIS II-FTIR (4000–400 cm^-1^) as KBr pellets. The number of protons was checked by means of thermogravimetris analysis (TGA) carried out on a Perkin-Elmer TGA/DSC instrument with a heating rate of 5 °C/min in flowing N_2_.

### 3.3. Polymerization procedure and polymer characterization

THF polymerization experiments were carried out in a stirred flask at 20 °C. Typically, a fixed amount of catalyst was added to the mixture of THF (10 mL) and acetic anhydride (2 mL) under stirring. Polymerization was terminated with the addition of saturated NaOH aqueous solution and then stirred for 5 min. At the end of the reaction, the precipitated polymer was filtered off, and then dissolved in butanone. After removing the catalyst by filtration, the polymer was precipitated in methanol for characterization and viscosimetric measurements. Intrinsic viscosity ([η]) of poly(THF) obtained were measured in tetrahydrofuran solution at 25 °C by using a Ubbelohde type viscometer. ^1^H-NMR spectra were recorded on a Bruker Avance 400 MHz spectrophotometer using 5 mm NMR tubes and deuterated acetone-D6 as solvent.

## 4. Conclusion

Bulk polymerization of THF was performed using H_3_PW_12_O_40_·13H_2_O as a solid acid catalyst. The effects of acetic anhydride concentration and catalyst amount were investigated. An increase in the AA and catalyst amounts led to a decrease in the viscosity of poly(THF). In the absence of AA, THF does not polymerize. The use of H_3_PW_12_O_40_ as a solid acid catalyst represents a more environmentally friendly alternative for the polymerization process. Such a catalyst offers many advantages compared to classical homogeneous catalysts: milder reaction conditions, easier separation of the catalyst from the reaction mixture by filtration, and its possible regeneration and reuse, reducing the production of waste and thus harm to the environment.

## Figures and Tables

**Table 1:**
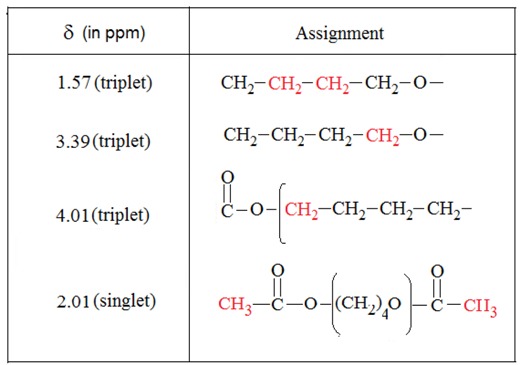
Chemical shift of polymer protons.

**Table 2:** Influence of the AA proportion on the intrinsic viscosity of poly(THF) catalyzed by 0.1g of H_3_PW_12_O_40_·13H_2_O. Reaction conditions: T = 20 °C; Reaction time = 1.5 h.

AA/THF (volume ratio)	[η] (dL g^-1^)
0.20	0.03899
0.25	0.03599
0.30	0.03499
0.40	0.02600
